# Cardiovascular, Kidney Failure, and All-Cause Mortality Events in Patients with FSGS in a US Real-World Database

**DOI:** 10.34067/KID.0000000000000469

**Published:** 2024-05-15

**Authors:** Juan Carlos Q. Velez, Kamlesh M. Thakker, Mark E. Bensink, Edgar V. Lerma, Richard Lieblich, C. Martin Bunke, Wu Gong, Kaijun Wang, Andrew R. Rava, Diana T. Amari, David Oliveri, Michael V. Murphy, David M.W. Cork

**Affiliations:** 1 Department of Nephrology, Ochsner Health, New Orleans, Louisiana; 2 Ochsner Clinical School, The University of Queensland, Brisbane, Queensland, Australia; 3 Notting Hill Consulting LLC, Celebration, Florida; 4 Travere Therapeutics, Inc., San Diego, California; 5 University of Illinois Chicago/Advocate Christ Medical Center, Oak Lawn, Illinois; 6 VJA Consulting, Walnut Creek, California; 7 CM Bunke Consulting, Mt. Pleasant, South Carolina; 8 Genesis Research Group, Hoboken, New Jersey; 9 Genesis Research Group, Newcastle upon Tyne, United Kingdom

**Keywords:** cardiovascular events, glomerular disease, glomerulosclerosis, kidney failure, mortality, nephrology, progression of renal failure

## Abstract

**Key Points:**

- In our patients with FSGS, elevated proteinuria and progression to kidney failure (KF) were associated with a higher risk of cardiovascular disease/all-cause mortality events.
- In addition, elevated pre-KF proteinuria was associated with KF/all-cause mortality events.
- CKD stage, nephrotic syndrome, and cardiovascular disease event rates, as well as the incremental costs of these events, were high.

**Background:**

FSGS leads to proteinuria and progressive decline in GFR, which correlates with kidney failure (KF) and increased cardiovascular risk. The purpose of this study was to estimate the effects of proteinuria on KF status/all-cause mortality and cardiovascular disease (CVD) events/all-cause mortality, as well as the relationship between progression to KF and occurrence of CVD/mortality events among adult patients (18 years or older) with FSGS.

**Methods:**

This was an observational, retrospective cohort study utilizing Optum deidentified Market Clarity Data and proprietary Natural Language Processing data. The study period was from January 1, 2007, through March 31, 2021, with patients in the overall cohort being identified from July 1, 2007, through March 31, 2021. The index date was the first FSGS ICD-10 diagnosis code or FSGS-related natural language processing term within the identification period.

**Results:**

Elevated proteinuria >1.5 and ≥3.5 g/g increased the risk of KF/all-cause mortality (adjusted hazard ratio [HR] [95% confidence interval (CI)], 2.34 [1.99 to 2.74] and 2.44 [2.09 to 2.84], respectively) and CVD/all-cause mortality (adjusted HR [95% CI], 2.11 [1.38 to 3.22] and 2.27 [1.44 to 3.58], respectively). Progression to KF was also associated with a higher risk of CVD/all-cause mortality (adjusted HR [95% CI], 3.04 [2.66 to 3.48]).

**Conclusions:**

A significant proportion of patients with FSGS experience KF and CVD events. Elevated proteinuria and progression to KF were associated with a higher risk of CVD/all-cause mortality events, and elevated pre-KF proteinuria was associated with progression to KF/all-cause mortality events. Treatments that meaningfully reduce proteinuria and slow the decline in GFR have the potential to reduce the risk of CVD, KF, and early mortality in patients with FSGS.

## Visual Abstract

**Figure:**
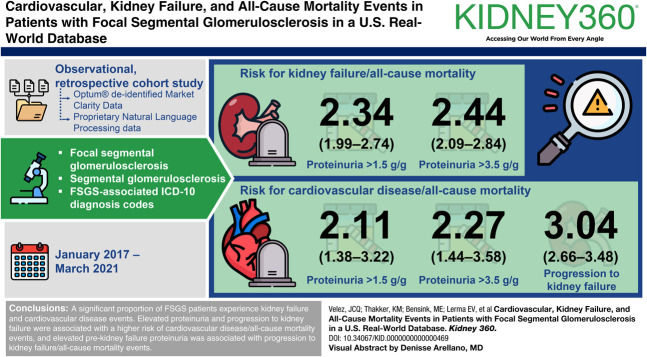

## Introduction

FSGS is a morphological pattern of glomerular damage characterized by the presence of sclerosis in specific segments of certain glomeruli. Persistent injury leads to proteinuria and a progressive decline in GFR.^1–3^ FSGS is classified into primary, genetic, secondary, and undetermined cause, differing in prognosis and management.^4^ Primary FSGS clinically manifests through nephrotic syndrome and is marked by substantial proteinuria, low serum albumin levels, hypertension, elevated cholesterol, and edema. Secondary FSGS arises from a range of underlying conditions or factors, including infection, drug toxicities, kidney maladaptation, and certain systemic diseases, such as obesity and sickle cell anemia.^5,6^ In addition, there are genetic forms of FSGS, which can be hereditary or appear sporadically, often presenting in childhood but also emerging in adulthood due to new mutations.^2^

FSGS is the most frequent glomerular disease leading to kidney failure (KF) in the United States^7^ despite its rarity, with an average annual estimated standardized prevalence (2016–2020) of 212.26 per 1,000,000 based on the US Census Bureau data.^8^ A diagnosis of FSGS can have a significant effect on a patient's quality of life, with high clinical and economic burden.^9–11^ Treatment is dependent on the type of FSGS; however, in all subtypes of FSGS, BP control is a key aspect of therapy, combining angiotensin-converting enzyme inhibitors or angiotensin receptor blockers with dietary sodium restriction. Primary FSGS is treated using immunosuppressive agents, such as prednisone, calcineurin inhibitors (CNIs), or mycophenolate mofetil plus high-dose dexamethasone.^3,7^ Patients with secondary FSGS receive disease-specific therapy other than immunosuppression.^12^

Although to date there is no US Food and Drug Administration–approved pharmacological treatment for FSGS, reduction in proteinuria is widely regarded to be beneficial in management of patients with FSGS and is considered the primary goal of treatment to slow the progressive course of the disease.^13^ For patients with FSGS, nephrotic syndrome is defined as proteinuria >3.5 g/g plus serum albumin <30 g/L, while patients with a spot urine protein-to-creatinine ratio (UPCR) ≥3.5 g/g and no hypoalbuminemia have nephrotic-range proteinuria.^4^

The relationship between proteinuria and kidney outcomes in FSGS is established, as a US-based cohort study, defining elevated proteinuria as ≥3 g/d, showed that biopsy-confirmed primary FSGS patients with elevated proteinuria have 10-year kidney survival rates of 57%, compared with 92% in those with lower levels of proteinuria (<3 g/d).^14^ In addition, reduction in nephrotic-range proteinuria to non-nephrotic levels (<3.5 g/d) is associated with significant improvement in kidney survival (hazard ratio [HR] [95% confidence intervals (CIs)], 0.48 [0.24 to 0.96]).^15^ While studies have been published evaluating kidney outcomes in FSGS patients with nephrotic syndrome, up-to-date analyses using robust methodology and a large sample of patients are needed.

Furthermore, cardiovascular risk increases with increasing levels of proteinuria and decreasing eGFR.^16^ The relative risk of cardiovascular disease (CVD) mortality associated with proteinuria ranges from 1.2 to 2.9.^17^ However, current publications evaluating CVD risk have assessed broader populations, not focusing solely on FSGS; limited studies are available analyzing the association between elevated proteinuria and risk of CVD/all-cause mortality and KF/all-cause mortality events, and between KF and CVD/all-cause mortality events in a US FSGS population.^18,19^

Therefore, the purpose of this study was to estimate the effects of proteinuria on KF status/all-cause mortality and CVD events/all-cause mortality, as well as the relationship between progression to KF and occurrence of CVD/all-cause mortality events among adult (18 years or older) patients with FSGS in the United States.

## Methods

### Study Design and Data Source

This was an observational, retrospective cohort study utilizing Optum deidentified Market Clarity Data and proprietary natural language processing (NLP) data. The Optum deidentified Market Clarity Data links electronic health record data from providers across the care continuum with historical, linked administrative claims data, pharmacy claims, and facility claims (with clinical information) and is inclusive of medications prescribed and administered. It is fully health insurance portability and accountability act-compliant, statistician-certified, deidentified data. The Optum NLP system was developed using vocabulary from the Unified Medical Language System that includes multiple medical dictionaries, such as the Logical Observation Identifiers Names and Codes, the Systemized Nomenclature of Medicine-Clinical Terms, and RxNorm, a listing of generic and branded drugs (among others). NLP concepts are identified and created based on broad topics, such as medications, signs, disease and symptoms, measurements, and observations. The data are harvested from the “Notes” fields within the Electronic Medical Records provided to Optum from over 50 large healthcare systems throughout the United States. The data used for development of each NLP concept are deidentified, and accuracy is verified through a series of quality assurance steps before release for use. Each NLP concept included in the data are associated with a unique subject record and a date of observation, allowing longitudinal tracking of concepts over time.

Institutional review board approval was not required for this study. The study period was from January 1, 2007, through March 31, 2021. Patients in the overall cohort were identified from July 1, 2007, through March 31, 2021, and patients in the incremental cost subcohort were identified from July 1, 2007, to September 30, 2020. The index date was defined as the date of the first FSGS international classification of diseases, tenth revision diagnosis code or FSGS-related NLP term within the identification period.

### Study Population

**Figure 1:**
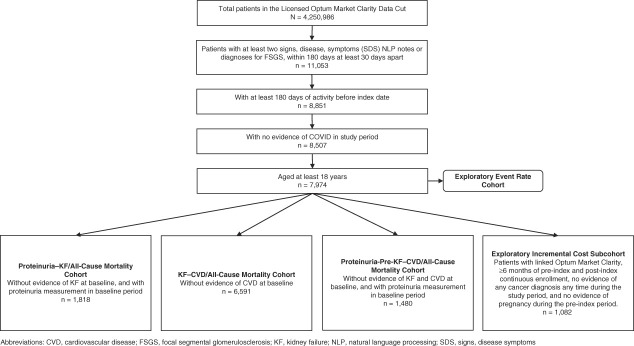
**Cohort attrition**. COVID, coronavirus disease; CVD, cardiovascular disease; KF, kidney failure; NLP, natural language processing; SDS, signs, disease, symptoms.

Owing to limited data available on the presence of a kidney biopsy, patients in the overall cohort were eligible if they were adults, aged 18 years or older, and had ≥2 signs, disease, symptoms (SDS) term entries with “focal segmental glomerulosclerosis” or “segmental glomerulosclerosis” and/or FSGS-associated ICD-10 diagnosis codes (N03.1, N04.1, N05.1, N06.1, N07.1) within 180 days total but at least 30 days apart (Figure 1). In addition, patients had to have ≥6 months before index continuous enrollment. It should be noted that these criteria precluded subtypes of FSGS to be distinguished from one another, so all subtypes may be represented in this analysis. Patients with negation terms in relation to FSGS NLP terms (*e.g*., negation terms will include “deny,” “failed,” “ignore,” “n/a,” “negative,” “question,” “reject,” “rule out,” “uncertain,” “unspecified”) were excluded from the study (Supplemental Figure 1). In addition, patients with evidence of coronavirus disease 2019 preindex or postindex were excluded to focus on clinical outcomes attributable to FSGS. The baseline period was defined as 6 months before index.

The overall cohort of eligible patients were classified into three groups to answer different research questions; a proteinuria–KF/all-cause mortality cohort used to examine the relationship between proteinuria and KF/all-cause mortality events, a KF–CVD/all-cause mortality cohort used to examine the relationship between KF status and CVD/all-cause mortality events, and a proteinuria-pre-KF–CVD/all-cause mortality cohort used to examine the relationship between pre-KF–proteinuria and CVD/all-cause mortality events. An additional, exploratory, incremental cost subcohort is discussed in the Supplemental Material section. In the proteinuria–KF/all-cause mortality cohort, patients were excluded if there was evidence of KF at baseline or no proteinuria measurement during the baseline period, while patients in the KF–CVD/all-cause mortality cohort were excluded if there was evidence of CVD at baseline (CVD event during baseline period or evidence of myocardial infarction, congestive heart failure [CHF], or stroke by the Charlson comorbidity index [CCI]). In the proteinuria-pre-KF–CVD/all-cause mortality cohort, patients were excluded if there was evidence of KF (based on diagnosis codes, eGFR <15 ml/min per 1.73 m^2^, procedure codes for dialysis or kidney transplant), evidence of CVD at baseline (as defined above), and if there was no proteinuria measurement during the baseline period.

### Variables and Outcomes

Information on the demographic and clinical characteristics included age, sex, region, race/ethnicity, insurance type, preindex enrollment, follow-up time, CCI, eGFR, UPCR, and CKD stage in the baseline period. The kidney and CVD outcome measures assessed during the follow-up period included number of events per patient, number of patients with an event, time to first event, and rate of events per 100 person-years.

Patients with KF events were defined as those with the occurrence of any of the components of KF, defined as CKD stage 5 (eGFR <15 ml/min per 1.73 m^2^ or diagnosis code [ICD-10: N18.5]), kidney transplant procedure, or dialysis (in-center hemodialysis, home hemodialysis, and peritoneal dialysis).

Patients with specific atherosclerotic CVD-related events were defined as those with ≥1 hospital admission with a primary diagnosis of myocardial infarction, unstable angina, ischemic stroke, transient ischemic attack, or CHF or ≥1 inpatient or outpatient revascularization procedure (percutaneous coronary intervention, coronary artery bypass graft).

All-cause mortality was defined as patients with a death date during follow-up, calculated as the last day of the month in which a patient died.

The effect of proteinuria was reported using adjusted HR with 95% CI for KF/all-cause mortality and CVD/all-cause mortality events. Elevated proteinuria was defined as UPCR >1.5 g/g^20^ or≥3.5 g/g^4^ with patients stratified by both UPCR ranges (≤1.5 versus >1.5 g/g and <3.5 versus ≥3.5 g/g). Adjusted HRs with 95% CI were also calculated for the association between KF status and CVD/all-cause mortality events.

### Statistical Analysis

Baseline demographic and clinical characteristics were analyzed descriptively, summarizing categorical variables using frequencies and percentages. Means with SDs and medians with quartile ranges were reported for continuous variables.

UPCRs were reported based on observed values or calculated for patients who had both a urine protein and urine creatinine value on the same day. Kaplan–Meier analysis and multivariate Cox proportional hazards models with time-dependent predictors were used to assess the association between baseline proteinuria and KF/all-cause mortality or CVD/all-cause mortality events and between baseline KF status and CVD/all-cause mortality events during follow-up. The time-dependent methodology allowed for all episodes for a given patient to be utilized in the models. The modeling analysis included patients with only baseline proteinuria; their proteinuria during follow-up was categorized based on their baseline values.

For the proteinuria and KF/all-cause mortality analysis, and the KF status and CVD/all-cause mortality analysis, censoring occurred at the end of database activity or end of the study period. For the proteinuria and CVD/all-cause mortality analysis, censoring occurred at the end of database activity, end of the study period, or transition to post-KF.

Univariate associations for all demographic, baseline health status, and baseline medication variables were tested for inclusion in the multivariable model. A Cox proportional hazards model adjusting for age, sex, diabetes, or peptic ulcer disease (CCI) and the use of diuretics, *β* blockers, or calcium channel blockers at baseline was used to determine the association between elevated proteinuria and CVD/all-cause mortality events in the follow-up period.

To determine the association between elevated proteinuria and KF/all-cause mortality events in the follow-up period, a Cox proportional hazards model was used, adjusting for age, insurance status, race/ethnicity, sex, baseline CKD stage, CHF, diabetes, dementia, or peptic ulcer disease (CCI) and the use of CNI, mycophenolate, *β* blockers, or calcium channel blockers at baseline.

For the KF status and CVD/all-cause mortality event analysis, a separate Cox proportional hazards model was used, adjusting for age, insurance status, sex, race/ethnicity, CCI comorbidities of cancer, peripheral vascular disease, HIV/AIDS, diabetes, chronic pulmonary disease, peptic ulcer disease, and hemiplegia/paraplegia and the use of diuretics, *β* blockers, calcium channel blockers, renin–angiotensin system inhibitors, CNIs, or mycophenolate at baseline.

Full details on the methods for the model establishment/variable selection, exploratory event rate, and cost analyses are included in the Supplemental Material.

Data analysis for this article was generated using SAS software (Copyright 2023 SAS Institute Inc.). SAS and all other SAS Institute Inc. product or service names are registered trademarks or trademarks of SAS Institute Inc., Cary, NC.

## Results

### Baseline and Demographic Characteristics

Overall, 7974 adult patients met the general inclusion criteria of whom 1818 (22.3%) met the criteria for inclusion in the proteinuria-KF/all-cause mortality cohort (Table 1). The mean age in this cohort was 52.0 years, 44.8% were female, and 57.2% were non-Hispanic White. The median (Q1–Q3) baseline UPCR was 2.3 (0.8–4.8) g/g. During follow-up, the mean number of proteinuria measurements was 5.2 and 22.2% of patients only had a measurement during baseline.

**Table 1:** Baseline demographic and clinical characteristics

Characteristic	Proteinuria–KF/All-Cause Mortality Cohort	KF–CVD/All-Cause Mortality Cohort	Proteinuria–Pre-KF–CVD/All-Cause Mortality Cohort
*N* (%)	1818	6591	1480
**Age, yr**			
Mean (SD)	52 0.0 (16.5)	49.5 (16.2)	49.9 (16.3)
Median (IQR)	53.0 (39.0–65.0)	50.0 (37.0–62.0)	51.0 (37.0–62.0)
**Age, *n* (%)**			
18–45	632 (34.8)	2679 (40.6)	590 (39.9)
46–65	726 (39.0)	2592 (39.3)	582 (39.3)
65+	460 (25.3)	1320 (20.0)	308 (20.8)
**Sex, *n* (%)**			
Female	814 (44.8)	2913 (44.2)	672 (45.4)
**Region, *n* (%)**			
Midwest	992 (54.6)	3167 (48.1)	807 (54.5)
Northeast	274 (15.1)	900 (13.7)	224 (15.1)
Other/unknown	74 (4.1)	296 (4.5)	57 (3.9)
South	257 (14.1)	1316 (20.0)	208 (14.1)
West	221 (12.2)	912 (13.8)	184 (12.4)
**Race/ethnicity, *n* (%)**			
Hispanic	166 (9.1)	585 (8.9)	136 (9.2)
Non-Hispanic Asian	59 (3.2)	211 (3.2)	56 (3.8)
Non-Hispanic Black	383 (21.1)	1574 (23.9)	311 (21.0)
Non-Hispanic White	1039 (57.2)	3391 (51.4)	832 (56.2)
Other/unknown	171 (9.4)	830 (12.6)	145 (9.8)
**Insurance type, *n* (%)**			
Commercial	866 (47.6)	3140 (47.6)	761 (51.4)
Medicaid	290 (16.0)	1052 (16.0)	233 (15.7)
Medicare	568 (31.2)	2021 (30.7)	404 (27.3)
Other payor type	23 (1.3)	116 (1.8)	22 (1.5)
Uninsured	61 (3.4)	174 (2.6)	52 (3.5)
Unknown	10 (0.6)	88 (1.3)	8 (0.5)
**CCI**			
Mean (SD)	2.0 (1.7)	1.2 (1.3)	1.6 (1.4)
Median (IQR)	1.0 (1.0–3.0)	1.0 (0.0–2.0)	1.0 (1.0–2.0)
**Baseline eGFR**			
With available data, *n* (%)	1794 (98.7)	3896 (59.2)	1459 (98.6)
*Mean (SD)*	51.9 (30.6)	45.3 (33.0)	54.6 (31.3)
*Median (IQR)*	43.5 (27.1–70.8)	37.3 (18.7–66.5)	47.1 (28.8–75.2)
**Baseline CKD stage, *n* (%)**			
With available eGFR/diagnosis data	1802 (99.1)	5112 (77.6)	1466 (99.1)
*Stage 1: eGFR >90 or CKD diagnosis*	226 (12.4)	509 (7.7)	214 (14.5)
*Stage 2: eGFR 60–89 or CKD diagnosis*	331 (18.2)	645 (9.8)	286 (19.3)
*Stage 3: eGFR 30–59 or CKD diagnosis*	722 (39.7)	1540 (23.4)	589 (39.8)
*Stage 4: eGFR 15–29 or CKD diagnosis*	523 (28.8)	993 (15.1)	377 (25.5)
*Stage 5: eGFR <15 or CKD diagnosis*	—	1425 (21.6)	—
*Unknown*	16 (0.9)	1479 (22.4)	14 (0.9)
Dialysis or renal transplant at baseline, *n* (%)	—	692 (10.5)	—
*With available stage data or baseline KF event*	1802 (99.1)	5113 (77.6)	1466 (99.1)
*Baseline KF (stage 5, dialysis, or renal transplant)*	—	1492 (29.2)	—
**Baseline protein creatinine ratio, g/g (includes calculated UPCR)**			
Tested^a^	1818 (100.0)	1480 (22.5)	1480 (100.0)
*Mean (SD)*	3.7 (4.5)	3.3 (4.0)	3.3 (4.0)
*Median (IQR)*	2.3 (0.8–4.8)	2.1 (0.7–4.3)	2.1 (0.7–4.3)

CCI, Charlson comorbidity index; CVD, cardiovascular disease; IQR, interquartile range; KF, kidney failure; UPCR, urine protein-to-creatinine ratio.

a Among pre–kidney failure at baseline.

The KF–CVD/all-cause mortality cohort included 6591 (82.7%) patients with a mean age of 49.5 years, 44.2% being female, and 51.4% being non-Hispanic White. The median (Q1–Q3) baseline UPCR was 2.1 (0.7–4.3) g/g, and 1492 (29.2%) patients were post-KF at baseline.

The proteinuria–pre-KF–CVD/all-cause mortality cohort included 1480 (18.6%) patients with a mean age of 49.9 years, with 45.4% being female and 56.2% being non-Hispanic White. The median (Q1–Q3) UPCR was 2.1 (0.7–4.3) g/g. In this cohort, 21.4% of patients only had a proteinuria measurement during baseline and the mean number of proteinuria measurements during follow-up was 5.4.

### Proteinuria Association with KF/All-Cause Mortality Events and CVD/All-Cause Mortality

**Figure 2:**
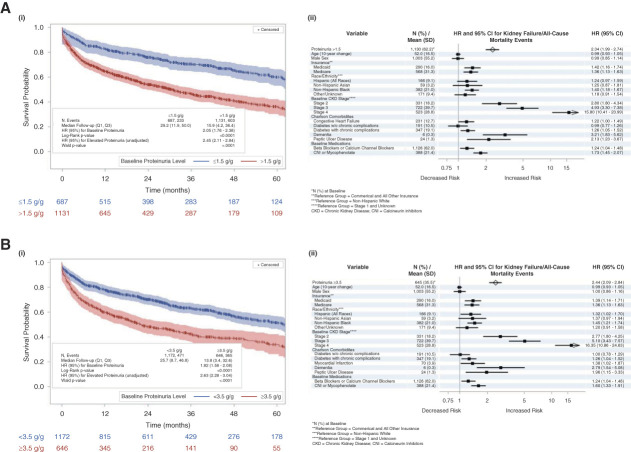
**Proteinuria association with KF/all-cause mortality events.** (A) Kaplan–Meier analysis (i) and Cox proportional model (ii) results for proteinuria and KF/all-cause mortality events by baseline proteinuria (1.5 g/g). (B) Kaplan–Meier analysis (i) and cox proportional model (ii) results for proteinuria and KF/all-cause mortality events by baseline proteinuria (3.5 g/g). Log-rank *P* value from Kaplan–Meier analysis, HR (95% CI) in Kaplan–Meier plot from univariate Cox proportional hazards model. Forest plot generated from the multivariable Cox proportional hazards model. Arrow at the end of the CI bar for metastatic solid tumor indicates the upper end of the CI extends beyond the *x* axis. CI, confidence interval; CNI, calcineurin inhibitor; HR, hazard ratio; Q1, first quartile; Q3, third quartile.

**Figure 3:**
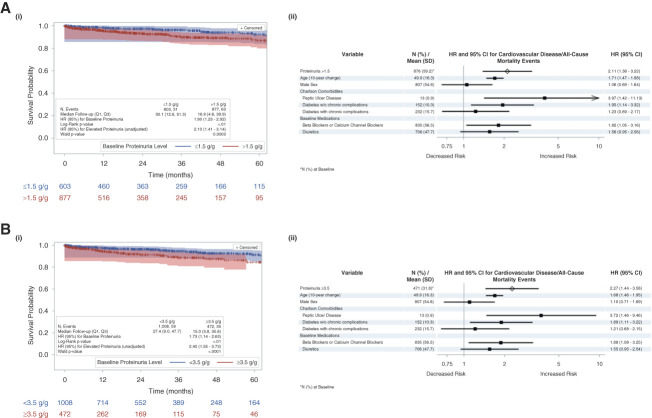
**Proteinuria association CVD/all-cause mortality events.** (A) Kaplan–Meier analysis (i) and Cox proportional model (ii) results for proteinuria and CVD/all-cause mortality events by baseline proteinuria (1.5 g/g). (B) Kaplan–Meier analysis (i) and cox proportional model (ii) results for proteinuria and CVD/all-cause mortality events by baseline proteinuria (3.5 g/g). Log-rank *P* value from Kaplan–Meier analysis, HR (95% CI) in Kaplan–Meier plot from univariate Cox proportional hazards model. Forest plot generated from the multivariable Cox proportional hazards model. Arrow at the end of the CI bar for metastatic solid tumor indicates the upper end of the CI extends beyond the *x* axis.

The Kaplan–Meier analysis showed a significantly higher risk of KF/all-cause mortality (*P* < 0.001) and CVD/all-cause mortality (*P* < 0.01) for patients with proteinuria >1.5 versus ≤1.5 g/g at baseline (Figures 2 and 3 and Table 2). In addition, the Kaplan–Meier analysis showed a significantly higher risk of KF/all-cause mortality (*P* < 0.001) and CVD/all-cause mortality (*P* < 0.01) for patients with proteinuria ≥3.5 versus <3.5 g/g at baseline.

**Table 2:** Proteinuria association with kidney failure/all-cause mortality events and cardiovascular disease/all-cause mortality

Category	Baseline/Time-Varying UPCR (g/g)					
	≤1.5 g/g	>1.5 g/g	*P* Value	<3.5 g/g	≥3.5 g/g	*P* Value
**Proteinuria**–**KF/all-cause mortality (Q1–Q3):**						
Median follow-up: months (range)	29.2 (11.9–50.0)	15.9 (4.2–36.4)		25.7 (8.7–46.8)	13.8 (3.4–32.6)	
KF/all-cause mortality events: *n*/*N* (%)	233/687 (33.9)	603/1131 (53.3)	<0.001	471/1172 (40.1)	365/646 (56.5)	<0.001
Unadjusted Cox HR (95% CI)		2.45 (2.11 to 2.84)	<0.001		2.63 (2.28 to 3.04)	<0.001
Adjusted Cox HR (95% CI)		2.34 (1.99 to 2.74)	—		2.44 (2.09 to 2.84)	—
**Proteinuria-CVD/all-cause mortality (Q1–Q3)**						
Median follow-up: months (range)	30.1 (12.6–51.3)	16.9 (4.6–39.9)		27.4 (9.0–47.7)	15.0 (3.8–35.6)	
KF/all-cause mortality events: *n*/*N* (%)	31/603 (5.1)	63/877 (7.2)	<0.01	59/1008 (5.9)	35/472 (7.4)	<0.001
Unadjusted Cox HR (95% CI)		2.10 (1.41 to 3.14)	<0.001		2.40 (1.55 to 3.73)	<0.001
Adjusted Cox HR (95% CI)		2.11 (1.38 to 3.22)	—		2.27 (1.44 to 3.58)	—

CI, confidence interval; CVD, cardiovascular disease; HR, hazard ratio; KF, kidney failure; UPCR, urine protein-to-creatinine ratio.

Both UPCR thresholds were associated with a statistically significant elevated risk of KF/all-cause mortality (>1.5 versus ≤1.5 g/g adjusted HR [95% CI], 2.34 [1.99 to 2.74]; and ≥3.5 versus <3.5 g/g adjusted HR [95% CI], 2.44 [2.09 to 2.84]) and CVD/all-cause mortality (>1.5 versus ≤1.5 g/g adjusted HR [95% CI], 2.11 [1.38 to 3.22]; and ≥3.5 versus <3.5 g/g adjusted HR [95% CI], 2.27 [1.44 to 3.58]).

### KF Status and CVD/All-Cause Mortality Events

**Figure 4:**
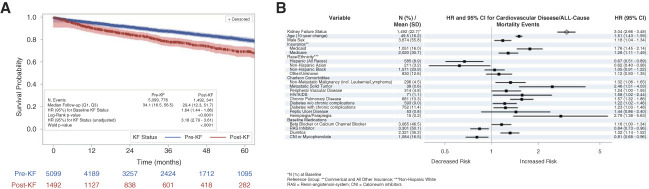
**KF status and CVD/all-cause mortality events.** Kaplan–Meier analysis (A) and Cox proportional model (B) results for CVD/all-cause mortality events by baseline KF status. Log-rank *P* value from Kaplan–Meier analysis, HR (95% CI) in Kaplan–Meier plot from univariate cox proportional hazards model. Forest plot generated from the multivariable cox proportional hazards model. Arrow at the end of the CI bar for metastatic solid tumor indicates the upper end of the CI extends beyond the *x* axis. RAS, renin–angiotensin system.

In the KF–CVD/all-cause mortality cohort, 776 of 5099 (15.2%) patients with baseline pre-KF status experienced a CVD/all-cause mortality event over a median (Q1–Q3) follow-up of 34.1 (16.5–56.5) months, while 341 of 1492 (22.9%) patients with baseline post-KF status experienced a CVD/all-cause mortality event over a median (Q1–Q3) follow-up of 29.4 (12.3–51.7) months (Figure 4). The Kaplan–Meier analysis showed a significantly higher risk of CVD/all-cause mortality for patients with baseline post-KF status (*P* < 0.001). In the unadjusted Cox proportional hazards model, post-KF status was associated with elevated risk of CVD/all-cause mortality events (HR [95% CI], 3.18 [2.79 to 3.61]; *P* < 0.001). In the time-dependent analysis (adjusted Cox proportional hazards model), after adjusting for covariates, post-KF status remained associated with a statistically significant elevated risk of CVD/all-cause mortality events (HR [95% CI], 0.04 [2.66 to 3.48]). Non-Hispanic Black patients did not have a statistically elevated risk compared with non-Hispanic White patients (HR [95% CI], 1.05 [0.91 to 1.22]). Hispanic patients had lower risk than non-Hispanic Black patients, with no overlap in 95% CIs, while non-Hispanic Asian patients also had lower risk than non-Hispanic Black patients, with slight overlap in 95% CIs.

### Exploratory Analysis

CKD stage progression, CVD events, and nephrotic syndrome events were associated with substantial incremental costs (Supplemental Tables 1 and 2). Patients with elevated baseline proteinuria (≥1.5, ≥3.5 g/g) and patients with KF at baseline experienced a high rate of CVD events per 100 person-years (Supplemental Table 3).

## Discussion

We believe that this is the first real-world study analyzing the effect of proteinuria on the risk of CVD/mortality events in patients with FSGS in the United States. The results demonstrated that elevated proteinuria evaluated by clinically relevant thresholds (>1.5, ≥3.5 g/g) and progression to KF were associated with significantly higher risk of experiencing a CVD/all-cause mortality event. In addition, we showed that pre-KF elevated proteinuria (>1.5, ≥3.5 g/g) was associated with a significantly elevated risk of progression to KF or all-cause mortality. The prognostic effects of proteinuria were consistently demonstrated through several approaches, including the Kaplan–Meier analysis by baseline proteinuria, univariate Cox models by time-dependent proteinuria, and the Cox model by time-dependent proteinuria adjusting for other risk factors, such as CKD stage, age, sex, and race. By utilizing time-dependent proteinuria, we ensured a comprehensive and dynamic reflection of disease progression through a validated metric accounting for the prognostic effects of proteinuria.^21^

In our exploratory analyses, CKD stage progression, the occurrence of CVD events, and the occurrence of nephrotic syndrome events were associated with substantial incremental costs. In addition, patients with elevated baseline proteinuria (≥1.5, ≥3.5 g/g) and patients with KF at baseline experienced a high rate of CVD events per 100 person-years.

Our US cohort study aligned with previous observational studies conducted in Canada and the United States, which have also demonstrated the clinical burden among patients with glomerular diseases.^18,19^ The US-based study, however, was not specific to FSGS, while the Canada-based study did not assess the effect of proteinuria on CVD and KF outcomes.^18,19^ Our study provides new insights into the relationship between proteinuria, KF, and CVD among a large sample of real-world patients with FSGS and, when included with the existing literature, shows that patients with FSGS experience an array of adverse KF and CVD outcomes, and underscores the particularly high burden of FSGS on patients.

This analysis is subject to limitations including potential errors in coding, incomplete or missing data, and patient visits to facilities that are not part of the integrated data network captured by Optum. Furthermore, patients were identified using diagnosis codes and NLP terms; therefore, they may not all be biopsy-proven FSGS. Errors in detection of FSGS-related terms in patient records may also introduce bias. In addition, this study did not differentiate between primary FSGS and other forms of FSGS, and the results of this study may not be applicable to other payer populations. It is possible that the post-KF event rates of patients are underestimated as patients may have transitioned from commercial to traditional Medicare coverage. Compared with the reports using different data sources, Hispanic and Asian patients appear to be underrepresented in this cohort.^10,11^ The cause of death is not included in the dataset; thus, we were not able to separate non-CVD and non-KF death from our all-cause mortality event outcomes. There are also limited data on BP, which is an independent risk factor for our outcomes. Finally, Optum uses standard pricing algorithms to account for variations in pricing across health plans and provider contracts, and therefore, the resulting cost information is meant to reflect allowed payments for all provider services across regions, and this cost information does not include out-of-pocket costs incurred by patients.

Using a robust methodology in a large sample of US patients with FSGS, our study showed that a significant proportion of patients with FSGS experience KF and CVD events. Our results also show that elevated proteinuria and progression to KF were associated with a higher risk of CVD and all-cause mortality events, and elevated pre-KF proteinuria was associated with progression to KF and all-cause mortality events. Treatments that meaningfully reduce proteinuria and slow the decline in eGFR have the potential to help reduce the risk of CVD, KF, and early mortality in patients with FSGS.

## Supplementary Material

## Data Availability

Partial restrictions to the data and/or materials apply. Deidentified data were used under Travere license agreement with Optum Market Clarity and are not publicly available. Data are available with permission from Optum Market Clarity.

## Supplemental Material

Supplemental Text

Supplemental Figure 1. Example attrition scenarios for SDS negation terms.

Supplemental Table 1. Baseline patient demographics for the exploratory incremental cost subcohort.

Supplemental Table 2. Linear regression analysis of incremental cost associated with CKD stage, nephrotic syndrome events and cerebrocardiovascular disease (CVD) events in adults.

Supplemental Table 3. Estimated CVD and nephrotic syndrome event rate in adults.

## References

[1] Abbate M Zoja C Remuzzi G. How does proteinuria cause progressive renal damage? J Am Soc Nephrol. 2006;17(11):2974–2984. doi:10.1681/ASN.2006040377 17035611

[2] De Vriese AS Sethi S Nath KA Glassock RJ Fervenza FC. Differentiating primary, genetic, and secondary FSGS in adults: a clinicopathologic approach. J Am Soc Nephrol. 2018;29(3):759–774. doi:10.1681/ASN.2017090958 29321142 PMC5827609

[3] Rosenberg AZ Kopp JB. Focal segmental glomerulosclerosis. Clin J Am Soc Nephrol. 2017;12(3):502–517. doi:10.2215/CJN.05960616 28242845 PMC5338705

[4] Kidney Disease: Improving Global Outcomes KDIGO Glomerular Diseases Work Group. KDIGO 2021 clinical practice guideline for the management of glomerular diseases. Kidney Int. 2021;100(4S):S1–S276. doi:10.1016/j.kint.2021.05.021 34556256

[5] Kim JS Han BG Choi SO Cha SK. Secondary focal segmental glomerulosclerosis: from podocyte injury to glomerulosclerosis. Biomed Res Int. 2016;2016:1630365. doi:10.1155/2016/1630365 27088082 PMC4819087

[6] Mayo Clinic. Focal Segmental Glomerulosclerosis (FSGS); 2023. Accessed November 15, 2023. https://www.mayoclinic.org/diseases-conditions/fsgs/symptoms-causes/syc-20354693

[7] Shabaka A Tato Ribera A Fernández-Juárez G. Focal segmental glomerulosclerosis: state-of-the-art and clinical perspective. Nephron. 2020;144(9):413–427. doi:10.1159/000508099 32721952

[8] Bensink ME Thakker K Lerma E, . EE265 focal segmental glomerulosclerosis (FSGS) in adults: a retrospective analysis of US prevalence and impacts of proteinuria and kidney function decline on healthcare resource utilization (HRU) and costs. Value Health. 2022;25(7):S385–S386. doi:10.1016/j.jval.2022.04.512

[9] Canetta PA Troost JP Mahoney S, . Health-related quality of life in glomerular disease. Kidney Int. 2019;95(5):1209–1224. doi:10.1016/j.kint.2018.12.018 30898342 PMC6743723

[10] Troost JP Waldo A Carlozzi NE, . The longitudinal relationship between patient-reported outcomes and clinical characteristics among patients with focal segmental glomerulosclerosis in the Nephrotic Syndrome Study Network. Clin Kidney J. 2020;13(4):597–606. doi:10.1093/ckj/sfz092 32905199 PMC7467600

[11] Kalantar-Zadeh K Baker CL Copley JB, . A retrospective study of clinical and economic burden of focal segmental glomerulosclerosis (FSGS) in the United States. Kidney Int Rep. 2021;6(10):2679–2688. doi:10.1016/j.ekir.2021.07.030 34622107 PMC8484118

[12] Korbet SM. Treatment of primary FSGS in adults. J Am Soc Nephrol 2012;23(11):1769–1776. doi:10.1681/ASN.2012040389 22997260

[13] D'Agati VD Kaskel FJ Falk RJ. Focal segmental glomerulosclerosis. N Engl J Med. 2011;365(25):2398–2411. doi:10.1056/NEJMra1106556 22187987

[14] Rydel JJ Korbet SM Borok RZ Schwartz MM. Focal segmental glomerular sclerosis in adults: presentation, course, and response to treatment. Am J Kidney Dis. 1995;25(4):534–542. doi:10.1016/0272-6386(95)90120-5 7702047

[15] Troyanov S Wall CA Miller JA Scholey JW Cattran DC; Toronto Glomerulonephritis Registry Group. Focal and segmental glomerulosclerosis: definition and relevance of a partial remission. J Am Soc Nephrol. 2005;16(4):1061–1068. doi:10.1681/ASN.2004070593 15716334

[16] Astor BC Coresh J Heiss G Pettitt D Sarnak MJ. Kidney function and anemia as risk factors for coronary heart disease and mortality: the Atherosclerosis Risk in Communities (ARIC) Study. Am Heart J. 2006;151(2):492–500. doi:10.1016/j.ahj.2005.03.055 16442920

[17] Currie G Delles C. Proteinuria and its relation to cardiovascular disease. Int J Nephrol Renovasc Dis. 2013;7:13–24. doi:10.2147/ijnrd.S40522 24379690 PMC3873205

[18] Canney M Gunning HM Zheng Y, . The risk of cardiovascular events in individuals with primary glomerular diseases. Am J Kidney Dis. 2022;80(6):740–750. doi:10.1053/j.ajkd.2022.04.005 35659570

[19] Go AS Tan TC Chertow GM, . Primary nephrotic syndrome and risks of ESKD, cardiovascular events, and death: the kaiser permanente nephrotic syndrome study. J Am Soc Nephrol. 2021;32(9):2303–2314. doi:10.1681/ASN.2020111583 34362836 PMC8729848

[20] Troost JP Trachtman H Nachman PH, . An outcomes-based definition of proteinuria remission in focal segmental glomerulosclerosis. Clin J Am Soc Nephrol. 2018;13(3):414–421. doi:10.2215/CJN.04780517 29167190 PMC5967666

[21] Barbour SJ Cattran DC Espino-Hernandez G Hladunewich MA Reich HN. Identifying the ideal metric of proteinuria as a predictor of renal outcome in idiopathic glomerulonephritis. Kidney Int. 2015;88(6):1392–1401. doi:10.1038/ki.2015.241 26287314

