# Supplementary material for: Cardiovascular, Kidney Failure, and All-Cause Mortality Events in Patients with FSGS in a US Real-World Database
Source: Kidney360. 2024 May 15;5(8):1145–53. doi: 10.34067/KID.0000000000000469 (PMC11371351; doi:10.34067/KID.0000000000000469)
Supplement: Supplementary file 1 [file kidney360-5-1145-s001.pdf]

ASN American Society of Nephrology JASN Journal of the American Society of Nephrology KIDNEY360 Accessing Our World From Every Angle CJASN Clinical Journal of the American Society of Nephrology

ASN Journal Disclosure Form

As per ASN journal policy, I have disclosed any financial relationship or commitment held by myself and/or my spouse/partner in the past 36 months as included below. I have listed my Current Employer below to indicate there is a relationship requiring disclosure. If no relationship exists, my Current Employer is not listed.

D. Amari reports the following:
Employer: Genesis Research Group; Ownership Interest: Genesis Research Group; and Other Interests or Relationships: I am an employee of Genesis Research Group, which was compensated by Travere Therapeutics for supporting this research.

I understand that the information above will be published within the journal article, if accepted, and that failure to comply and/or to accurately and completely report the potential financial conflicts of interest could lead to the following: 1) Prior to publication, article rejection, or 2) Post-publication, sanctions ranging from, but not limited to, issuing a correction, reporting the inaccurate information to the authors' institution, banning authors from submitting work to ASN journals for varying lengths of time, and/or retraction of the published work.

Name: Diana T Amari
Manuscript ID: K360-2024-000075R1
Manuscript Title: Cardiovascular, Kidney Failure, and All-Cause Mortality Events in Patients with Focal Segmental Glomerulosclerosis in a U.S. Real-World Database
Date of Completion: March 28, 2024
Disclosure Updated Date: December 1, 2023

ASN
American Society of Nephrology
JASN
Journal of the American Society of Nephrology
KIDNEY360
Accessing Our World From Every Angle
CJASN
Clinical Journal of the American Society of Nephrology

ASN Journal Disclosure Form

As per ASN journal policy, I have disclosed any financial relationship or commitment held by myself and/or my spouse/partner in the past 36 months as included below. I have listed my Current Employer below to indicate there is a relationship requiring disclosure. If no relationship exists, my Current Employer is not listed.

M. Bensink reports the following:
Employer: Benofit Consulting; Consultancy: Travere Therapeutics, Amgen; Ownership Interest: Amgen; and Research Funding: Travere Therapeutics, Amgen.

I understand that the information above will be published within the journal article, if accepted, and that failure to comply and/or to accurately and completely report the potential financial conflicts of interest could lead to the following: 1) Prior to publication, article rejection, or 2) Post-publication, sanctions ranging from, but not limited to, issuing a correction, reporting the inaccurate information to the authors' institution, banning authors from submitting work to ASN journals for varying lengths of time, and/or retraction of the published work.

Name: Mark Eliot Bensink
Manuscript ID: K360-2024-000075R1
Manuscript Title: Cardiovascular, Kidney Failure, and All-Cause Mortality Events in Patients with Focal Segmental Glomerulosclerosis in a U.S. Real-World Database
Date of Completion: March 26, 2024
Disclosure Updated Date: March 26, 2024

ASN American Society of Nephrology
JASN Journal of the American Society of Nephrology
KIDNEY360 Accessing Our World From Every Angle
CJASN Clinical Journal of the American Society of Nephrology

ASN Journal Disclosure Form

As per ASN journal policy, I have disclosed any financial relationships or commitments I have held in the past 36 months as included below. I have listed my Current Employer below to indicate there is a relationship requiring disclosure. If no relationship exists, my Current Employer is not listed.

M. Bunke reports the following:
Employer: C M Bunke Consulting LLC; Consultancy: Travere Therapeutics; and Ownership Interest: Travere Therapeutics.

I understand that the information above will be published within the journal article, if accepted, and that failure to comply and/or to accurately and completely report the potential financial conflicts of interest could lead to the following: 1) Prior to publication, article rejection, or 2) Post-publication, sanctions ranging from, but not limited to, issuing a correction, reporting the inaccurate information to the authors' institution, banning authors from submitting work to ASN journals for varying lengths of time, and/or retraction of the published work.

Name: Martin C. Bunke
Manuscript ID: K360-2024-000075R1
Manuscript Title: Cardiovascular, Kidney Failure, and All-Cause Mortality Events in Patients with Focal Segmental Glomerulosclerosis in a U.S. Real-World Database
Date of Completion: April 30, 2024
Disclosure Updated Date: December 1, 2023

ASN American Society of Nephrology
JASN Journal of the American Society of Nephrology
KIDNEY360 Accessing Our World From Every Angle
CJASN Clinical Journal of the American Society of Nephrology

ASN Journal Disclosure Form

As per ASN journal policy, I have disclosed any financial relationship or commitment held by myself and/or my spouse/partner in the past 36 months as included below. I have listed my Current Employer below to indicate there is a relationship requiring disclosure. If no relationship exists, my Current Employer is not listed.

D. Cork reports the following:
Employer: Putnam; Genesis Research

I understand that the information above will be published within the journal article, if accepted, and that failure to comply and/or to accurately and completely report the potential financial conflicts of interest could lead to the following: 1) Prior to publication, article rejection, or 2) Post-publication, sanctions ranging from, but not limited to, issuing a correction, reporting the inaccurate information to the authors' institution, banning authors from submitting work to ASN journals for varying lengths of time, and/or retraction of the published work.

Name: David M.w. Cork
Manuscript ID: K360-2024-000075R1
Manuscript Title: Cardiovascular, Kidney Failure, and All-Cause Mortality Events in Patients with Focal Segmental Glomerulosclerosis in a U.S. Real-World Database
Date of Completion: March 28, 2024
Disclosure Updated Date: December 14, 2023

ASN American Society of Nephrology | JASN Journal of the American Society of Nephrology | KIDNEY360 Accessing Our World From Every Angle | CJASN Clinical Journal of the American Society of Nephrology

ASN Journal Disclosure Form

As per ASN journal policy, I have disclosed any financial relationship or commitment held by myself and/or my spouse/partner in the past 36 months as included below. I have listed my Current Employer below to indicate there is a relationship requiring disclosure. If no relationship exists, my Current Employer is not listed.

E. Lerma reports the following:
Employer: Associates in Nephrology; Consultancy: Akebia, Astra Zeneca, Bayer, Boehringer Ingelheim, Calliditas, Glaxo Smith Kline, Novartis, Otsuka, Travere, Vifor; Ownership Interest: Fresenius Joint Venture; Honoraria: Honoraria for Advisory Board/ Speaker bureau: Akebia, Astra Zeneca, Bayer, Boehringer Ingelheim, Glaxo Smith Kline, Otsuka, Travere, Vifor; Patents or Royalties: UpToDate, McGraw-Hill Publishing, Elsevier Publishing, Wolters Kluwer Publishing, Springer; Advisory or Leadership Role: Editorial Board member: Clinical Journal of the American Society of Nephrology, Journal of Clinical Lipidology, International Urology and Nephrology Journal, Journal of Vascular Access, Prescribers Letter, Renal and Urology News, ASN Kidney News, Reviews in Endocrinology and Metabolic Disorders, American Journal of Kidney Diseases; and Speakers Bureau: Akebia, Astra Zeneca, Bayer, Boehringer Ingelheim, Glaxo Smith Kline, Otsuka, Travere, Vifor.

I understand that the information above will be published within the journal article, if accepted, and that failure to comply and/or to accurately and completely report the potential financial conflicts of interest could lead to the following: 1) Prior to publication, article rejection, or 2) Post-publication, sanctions ranging from, but not limited to, issuing a correction, reporting the inaccurate information to the authors' institution, banning authors from submitting work to ASN journals for varying lengths of time, and/or retraction of the published work.

Name: Edgar V. Lerma
Manuscript ID: K360-2024-000075R1
Manuscript Title: Cardiovascular, Kidney Failure, and All-Cause Mortality Events in Patients with Focal Segmental Glomerulosclerosis in a U.S. Real-World Database
Date of Completion: March 28, 2024
Disclosure Updated Date: March 28, 2024

ASN American Society of Nephrology JASN Journal of the American Society of Nephrology KIDNEY360 Accessing Our World From Every Angle CJASN Clinical Journal of the American Society of Nephrology

ASN Journal Disclosure Form

As per ASN journal policy, I have disclosed any financial relationship or commitment held by myself and/or my spouse/partner in the past 36 months as included below. I have listed my Current Employer below to indicate there is a relationship requiring disclosure. If no relationship exists, my Current Employer is not listed.

R. Lieblich reports the following:
Employer: VJA Consulting; Consultancy: Travere; Research Funding: Travere; and Honoraria: Travere.

I understand that the information above will be published within the journal article, if accepted, and that failure to comply and/or to accurately and completely report the potential financial conflicts of interest could lead to the following: 1) Prior to publication, article rejection, or 2) Post-publication, sanctions ranging from, but not limited to, issuing a correction, reporting the inaccurate information to the authors' institution, banning authors from submitting work to ASN journals for varying lengths of time, and/or retraction of the published work.

Name: Richard M. Lieblich
Manuscript ID: K360-2024-000075R1
Manuscript Title: "Cardiovascular, Kidney Failure, and All-Cause Mortality Events in Patients with Focal Segmental Glomerulosclerosis in a U.S. Real-World Database,"
Date of Completion: March 26, 2024
Disclosure Updated Date: December 14, 2023

ASN
American Society of Nephrology
JASN
Journal of the American Society of Nephrology
KIDNEY360
Accessing Our World From Every Angle
CJASN
Clinical Journal of the American Society of Nephrology

ASN Journal Disclosure Form

As per ASN journal policy, I have disclosed any financial relationship or commitment held by myself and/or my spouse/partner in the past 36 months as included below. I have listed my Current Employer below to indicate there is a relationship requiring disclosure. If no relationship exists, my Current Employer is not listed.

M. Murphy reports the following:
Employer: Genesis Research Group; and Other Interests or Relationships: I am an employee of Genesis Research Group, which was compensated by Travere Therapeutics for supporting this research.

I understand that the information above will be published within the journal article, if accepted, and that failure to comply and/or to accurately and completely report the potential financial conflicts of interest could lead to the following: 1) Prior to publication, article rejection, or 2) Post-publication, sanctions ranging from, but not limited to, issuing a correction, reporting the inaccurate information to the authors' institution, banning authors from submitting work to ASN journals for varying lengths of time, and/or retraction of the published work.

Name: Michael V Murphy
Manuscript ID: K360-2024-000075R1
Manuscript Title: Cardiovascular, Kidney Failure, and All-Cause Mortality Events in Patients with Focal Segmental Glomerulosclerosis in a U.S. Real-World Database
Date of Completion: March 26, 2024
Disclosure Updated Date: December 1, 2023

ASN American Society of Nephrology JASN Journal of the American Society of Nephrology KIDNEY360 Accessing Our World From Every Angle CJASN Clinical Journal of the American Society of Nephrology

ASN Journal Disclosure Form

As per ASN journal policy, I have disclosed any financial relationship or commitment held by myself and/or my spouse/partner in the past 36 months as included below. I have listed my Current Employer below to indicate there is a relationship requiring disclosure. If no relationship exists, my Current Employer is not listed.

D. Oliveri reports the following:
Employer: Genesis Research Group; Consultancy: I am an employee of Genesis Research Group, which was compensated by Travere Therapeutics for supporting this research.; and Ownership Interest: Genesis Research Group.

I understand that the information above will be published within the journal article, if accepted, and that failure to comply and/or to accurately and completely report the potential financial conflicts of interest could lead to the following: 1) Prior to publication, article rejection, or 2) Post-publication, sanctions ranging from, but not limited to, issuing a correction, reporting the inaccurate information to the authors' institution, banning authors from submitting work to ASN journals for varying lengths of time, and/or retraction of the published work.

Name: David Oliveri
Manuscript ID: K360-2024-000075R1
Manuscript Title: Cardiovascular, Kidney Failure, and All-Cause Mortality Events in Patients with Focal Segmental Glomerulosclerosis in a U.S. Real-World Database
Date of Completion: March 26, 2024
Disclosure Updated Date: December 6, 2023

ASN American Society of Nephrology | JASN Journal of the American Society of Nephrology | KIDNEY360 Accessing Our World From Every Angle | CJASN Clinical Journal of the American Society of Nephrology

ASN Journal Disclosure Form

As per ASN journal policy, I have disclosed any financial relationship or commitment held by myself and/or my spouse/partner in the past 36 months as included below. I have listed my Current Employer below to indicate there is a relationship requiring disclosure. If no relationship exists, my Current Employer is not listed.

A. Rava reports the following:
Employer: Genesis Research Group; and Other Interests or Relationships: I am an employee of Genesis Research Group, which was compensated by Travere Therapeutics for supporting this research.

I understand that the information above will be published within the journal article, if accepted, and that failure to comply and/or to accurately and completely report the potential financial conflicts of interest could lead to the following: 1) Prior to publication, article rejection, or 2) Post-publication, sanctions ranging from, but not limited to, issuing a correction, reporting the inaccurate information to the authors' institution, banning authors from submitting work to ASN journals for varying lengths of time, and/or retraction of the published work.

Name: Andrew Rava
Manuscript ID: K360-2024-000075R1
Manuscript Title: Cardiovascular, Kidney Failure, and All-Cause Mortality Events in Patients with Focal Segmental Glomerulosclerosis in a U.S. Real-World Database
Date of Completion: March 26, 2024
Disclosure Updated Date: March 26, 2024

ASN American Society of Nephrology | JASN Journal of the American Society of Nephrology | KIDNEY360 Accessing Our World From Every Angle | CJASN Clinical Journal of the American Society of Nephrology

ASN Journal Disclosure Form

As per ASN journal policy, I have disclosed any financial relationship or commitment held by myself and/or my spouse/partner in the past 36 months as included below. I have listed my Current Employer below to indicate there is a relationship requiring disclosure. If no relationship exists, my Current Employer is not listed.

K. Thakker reports the following:
Employer: Notting Hill Consulting LLC; Consultancy: Travere Therapeutics; TrexBio, TrialSpark, Notting Hill Consulting LLC; AlphaSights ; GLG; IFAPP Academy;; and Ownership Interest: Very small positions ($3000 approx) Pfizer, Abbvie, Astra-Zeneca, Roche, Eli Lilly, Biogen, Merck, several technology and biotechnology stocks, and ETFs in all financial industry sectors.

I understand that the information above will be published within the journal article, if accepted, and that failure to comply and/or to accurately and completely report the potential financial conflicts of interest could lead to the following: 1) Prior to publication, article rejection, or 2) Post-publication, sanctions ranging from, but not limited to, issuing a correction, reporting the inaccurate information to the authors' institution, banning authors from submitting work to ASN journals for varying lengths of time, and/or retraction of the published work.

Name: Kamlesh M. Thakker
Manuscript ID: K360-2024-000075R1
Manuscript Title: Cardiovascular, Kidney Failure, and All-Cause Mortality Events in Patients with Focal Segmental Glomerulosclerosis in a U.S. Real-World Database
Date of Completion: March 28, 2024
Disclosure Updated Date: December 1, 2023

ASN
American Society of Nephrology
JASN
Journal of the American Society of Nephrology
KIDNEY360
Accessing Our World From Every Angle
CJASN
Clinical Journal of the American Society of Nephrology

ASN Journal Disclosure Form

As per ASN journal policy, I have disclosed any financial relationships or commitments I have held in the past 36 months as included below. I have listed my Current Employer below to indicate there is a relationship requiring disclosure. If no relationship exists, my Current Employer is not listed.

J. Velez reports the following:
Employer: Ochsner Health; Consultancy: Mallinckrodt Pharmaceuticals, Travere Therapeutics, Calliditas; Honoraria: Mallinckrodt Pharmaceuticals, Travere Therapeutics, Calliditas; Advisory or Leadership Role: Mallinckrodt Pharmaceuticals, Travere Therapeutics, Calliditas; and Speakers Bureau: Mallinckrodt Pharmaceuticals.

I understand that the information above will be published within the journal article, if accepted, and that failure to comply and/or to accurately and completely report the potential financial conflicts of interest could lead to the following: 1) Prior to publication, article rejection, or 2) Post-publication, sanctions ranging from, but not limited to, issuing a correction, reporting the inaccurate information to the authors' institution, banning authors from submitting work to ASN journals for varying lengths of time, and/or retraction of the published work.

Name: Juan Carlos Q. Velez
Manuscript ID: K360-2024-000075R1
Manuscript Title: Cardiovascular, Kidney Failure, and All-Cause Mortality Events in Patients with Focal Segmental Glomerulosclerosis in a U.S. Real-World Database
Date of Completion: April 29, 2024
Disclosure Updated Date: March 27, 2024

ASN American Society of Nephrology JASN Journal of the American Society of Nephrology KIDNEY360 Accessing Our World From Every Angle CJASN Clinical Journal of the American Society of Nephrology

ASN Journal Disclosure Form

As per ASN journal policy, I have disclosed any financial relationship or commitment held by myself and/or my spouse/partner in the past 36 months as included below. I have listed my Current Employer below to indicate there is a relationship requiring disclosure. If no relationship exists, my Current Employer is not listed.

K. Wang reports the following:
Employer: Travere; and Ownership Interest: Travere.

I understand that the information above will be published within the journal article, if accepted, and that failure to comply and/or to accurately and completely report the potential financial conflicts of interest could lead to the following: 1) Prior to publication, article rejection, or 2) Post-publication, sanctions ranging from, but not limited to, issuing a correction, reporting the inaccurate information to the authors' institution, banning authors from submitting work to ASN journals for varying lengths of time, and/or retraction of the published work.

Name: Kaijun Wang
Manuscript ID: K360-2024-000075R1
Manuscript Title: Cardiovascular, Kidney Failure, and All-Cause Mortality Events in Patients with Focal Segmental Glomerulosclerosis in a U.S. Real-World Database
Date of Completion: March 26, 2024
Disclosure Updated Date: March 26, 2024

ASN
American Society of Nephrology
JASN
Journal of the American Society of Nephrology
KIDNEY360
Accessing Our World From Every Angle
CJASN
Clinical Journal of the American Society of Nephrology

ASN Journal Disclosure Form

As per ASN journal policy, I have disclosed any financial relationships or commitments I have held in the past 36 months as included below. I have listed my Current Employer below to indicate there is a relationship requiring disclosure. If no relationship exists, my Current Employer is not listed.

W. Gong reports the following:
Employer: Travere Therapeutics; and Ownership Interest: Travere Therapeutics.

I understand that the information above will be published within the journal article, if accepted, and that failure to comply and/or to accurately and completely report the potential financial conflicts of interest could lead to the following: 1) Prior to publication, article rejection, or 2) Post-publication, sanctions ranging from, but not limited to, issuing a correction, reporting the inaccurate information to the authors' institution, banning authors from submitting work to ASN journals for varying lengths of time, and/or retraction of the published work.

Name: Wu Gong
Manuscript ID: K360-2024-000075R1
Manuscript Title: Cardiovascular, Kidney Failure, and All-Cause Mortality Events in Patients with Focal Segmental Glomerulosclerosis in a U.S. Real-World Database
Date of Completion: April 18, 2024
Disclosure Updated Date: April 18, 2024
