# Supplementary material for: Cardiovascular, Kidney Failure, and All-Cause Mortality Events in Patients with FSGS in a US Real-World Database
Source: Kidney360. 2024 May 15;5(8):1145–53. doi: 10.34067/KID.0000000000000469 (PMC11371351; doi:10.34067/KID.0000000000000469)
Supplement: Supplementary file 2 [file kidney360-5-1145-s002.pdf]

<u>Supplementary Material</u>

Supplemental Text

Supplemental Figure 1 – Example Attrition Scenarios for SDS negation terms

Supplemental Table 1 – Baseline Patient Demographics for the Exploratory Incremental Cost Sub-Cohort

Supplemental Table 2 – Linear Regression Analysis of Incremental Cost Associated with CKD Stage, Nephrotic Syndrome Events and Cerebrocardiovascular disease (CVD) Events in Adults

Supplemental Table 3 – Estimated CVD and Nephrotic Syndrome Event Rate in Adults

Supplemental Text

Model Establishment Methodology

The model establishment started with testing univariate associations for all demographics (age, gender, region, insurance type, index year), baseline health status (Charlson comorbidities, Charlson comorbidity index, CVD events, CKD stage) and baseline medications (antihypertensives, beta blockers, calcium channel blockers, mineralocorticoid receptor antagonists, immunosuppressives, calcineurin inhibitors, renin-angiotensin system inhibitors, potassium binders, mycophenolate, diuretics, statins, angiotensin-converting enzyme inhibitors, angiotensin II receptor blockers, sodium-glucose cotransporter-2 inhibitors, glucagon-like peptid-1 receptor antagonists, rituximab, and glucocorticoids) for inclusion in the multivariable model. Variables with a p-value <0.30 in the univariate results were considered for inclusion in the multivariable model.

Further, only the independent variables of interest were initially included, and variables were added to the model in a stepwise fashion. Variables were retained if they were statistically significant (p <0.05), if their addition improved the model's Akaike information criterion (AIC) or had an appreciable impact on the HR for the independent variable of interest. If the addition of a new variable caused a previously

retained variable to lose statistical significance, we tested removing the variable and assessed model AIC and HR for the variable of interest.

Exploratory Incremental Costs Analysis

Patients in the exploratory incremental cost sub-cohort were identified from July 1, 2007 to September 30, 2020, allowing for a 6 month follow-up and were required to have linked Optum® Market Clarity claims data, and ≥6 months of pre- and post-index continuous enrollment. Those with evidence of cancer or COVID-19 pre- or post-index, or pregnancy pre-index were excluded (**Figure 1**).

The incremental costs associated with having a CVD or KF event were estimated using a linear regression model, adjusting for age, Charlson Comorbidity Index (CCI), baseline CKD stage, gender, index year, insurance type, region, proteinuria events, steroid use events, and nephrotic-level proteinuria events. The log-transformed costs were modeled using a Gaussian distribution and identity link. Costs were re-transformed using a smear technique. All costs were adjusted to 2022 US dollars using the Consumer Price Index and presented as per-patient-per-month (PPPM) values.

Exploratory Event Rate Analysis

Patients in the exploratory event rate analysis were identified from July 1, 2007 to March 31, 2021, allowing for a 6 month baseline and were adult (≥18 years old) patients with at least two SDS NLP term entries for "focal_segmental_glomerulosclerosis" or "segmentalglomerulosclerosis" AND/OR FSGS-associated ICD-10 diagnosis codes (N03.1, N04.1, N05.1, N06.1, N07.1), within 180 days at least 30 days apart within the identification period. Patients with negation terms (e.g., 'deny', 'failed', 'ignore', 'n/a', 'negative', 'question', 'reject', 'rule out', 'uncertain', 'unspecified') in relation to the FSGS SDS term were excluded. Patients were required to have ≥6 months of pre-index activity (baseline period). The index date was the first FSGS ICD-10 diagnosis code or NLP term within the identification period. Patients with evidence of COVID-19 pre- or post-index were excluded (**Figure 1**).

Rates of CVD and nephrotic syndrome events were reported as events per 100 person years following the index date. Patients were followed from the index date to the end of EHR/claims activity or to the end date of study period. End of follow-up was the earliest of the end date of EHR/claims activity or end date of study period (March 31, 2021). CVD events were defined as patients with ≥1 hospital admission with primary diagnosis of myocardial infarction (MI), unstable angina, ischemic stroke, transient ischemic attack (TIA), or congestive heart failure or ≥1 inpatient or outpatient revascularization procedure (percutaneous coronary intervention [PCI], coronary artery bypass graft [CABG]). Nephrotic syndrome events were defined as patients with ≥1 UPCR ≥3.0 g/g or 24-hour urine protein ≥3.5 g/day and serum albumin levels <3.0 g/dL or with ≥1 hospital admission or outpatient visit with diagnosis associated with nephrotic syndrome.

Supplemental Figure 1: Example Attrition Scenarios for SDS negation terms

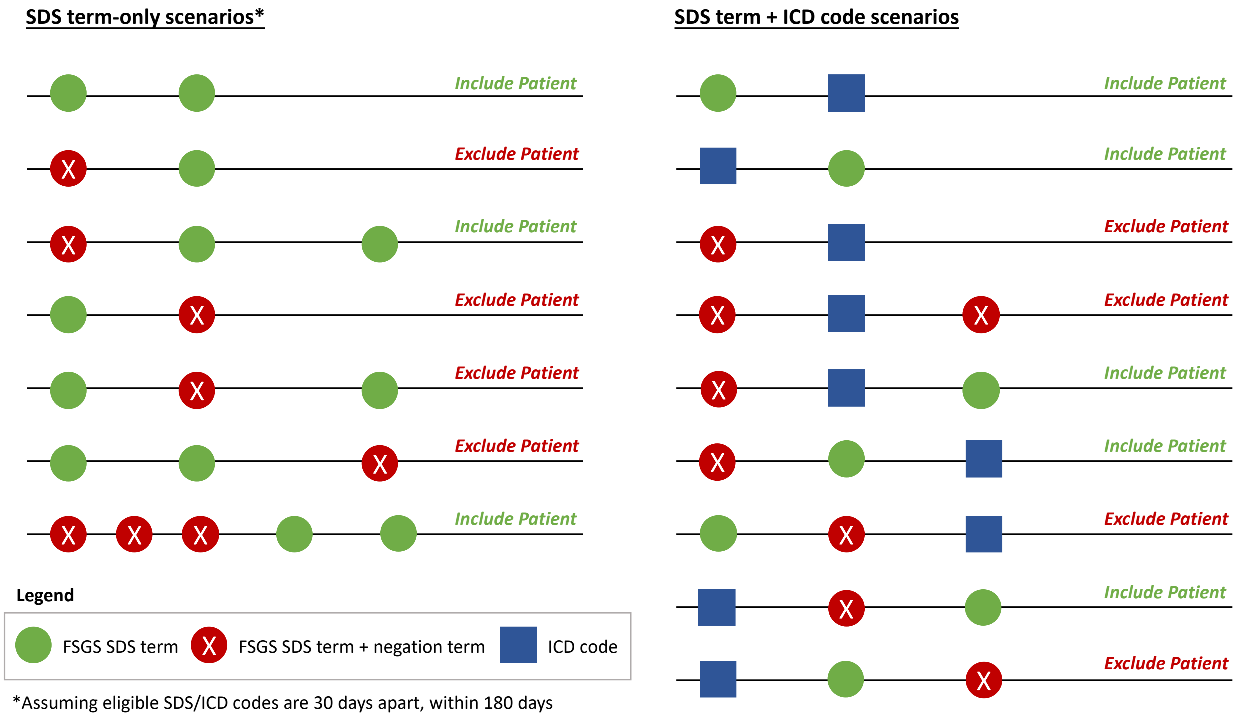

*Assuming eligible SDS/ICD codes are 30 days apart, within 180 days

Supplemental Table 1: Baseline Patient Demographics and Clinical Characteristics for the Exploratory Incremental Cost Sub-Cohort

Characteristics	Exploratory Incremental Cost Sub-Cohort (n=1,082)
**Age, years**	
Mean (SD)	50.7 (15.8)
Median (Q1-Q3)	51.0 (40.0 – 62.0)
**Age, n (%)**	
18-45 years	381 (35.2%)
46-65 years	499 (46.1%)
65+ years	202 (18.7%)
**Gender, n (%)**	
Female	464 (42.9%)
**Region, n (%)**	
Midwest	512 (47.3%)
Northeast	206 (19.0%)
Other/Unknown	54 (5.0%)
South	180 (16.6%)
West	130 (12.0%)
**Race/Ethnicity, n (%)**	
Hispanic (All Races)	85 (7.9%)
Non-Hispanic Asian	32 (3.0%)
Non-Hispanic Black	268 (24.8%)
Non-Hispanic White	547 (50.6%)
Other/Unknown	150 (13.9%)
**Insurance Type, n (%)**	
Commercial	584 (54.0%)
Medicaid	177 (16.4%)
Medicare	281 (26.0%)
Other Payor Type	3 (<1%)
Uninsured	15 (1.4%)
Unknown	22 (2.0%)
**Baseline eGFR, mL/min/1.73 m^2**	
With available data**, n (%)**	610 (56.4%)
Mean (SD)	42.8 (30.4)
Median (Q1-Q3)	36.0 (18.7 – 61.1)
**Baseline CKD stage, n (%)**	
With available data	900 (83.2%)
Stage 1: eGFR >90 or CKD diagnosis	68 (6.3%)
Stage 2: eGFR 60-89 or CKD diagnosis	104 (9.6%)
Stage 3: eGFR 30-59 or CKD diagnosis	294 (27.2%)
Stage 4: eGFR 15-29 or CKD diagnosis	185 (17.1%)
Stage 5: eGFR <15 or CKD diagnosis	249 (23.0%)
Unknown	182 (16.8%)
**Available CKD stage or KF data, n (%)**	900 (83.2%)
Baseline KF, n (%)	262 (29.1%)
Baseline dialysis or renal transplant, n (%)	168 (15.5%)
Stage 5: eGFR <15 or CKD diagnosis, n (%)	249 (27.7%)
**Baseline proteinuria, g/day**	
With available data, n (%)	271 (25.0%)
Mean (SD)	3.7 (4.1)
Median (Q1-Q3)	2.2 (0.8 – 5.4)
**CCI**	

Mean (SD)	1.6 (1.5)
Median (Q1-Q3)	1.0 (0.0 – 2.0)

Supplemental Table 2: Linear Regression Analysis of Incremental Cost Associated with CKD Stage, Nephrotic Syndrome Events and Cerebrocardiovascular disease (CVD) Events in Adults

Event	Incremental Cost (PPPM)	SD
CKD Stage 3	$1,758.97	$35,318.00
CKD Stage 4	$2,693.36	$48,033.30
CKD Stage 5	$7,315.30	$84,086.20
Nephrotic Syndrome Event	$4,069.19	$36,905.60
CVD Event	$7,104.59	$81,026.00

Incremental cost by CKD stage is based on average cost for CKD 1/2; $4,736.62
Baseline CKD stage, age, gender, region, insurance type, Charlson index, index year, CV event, Nephrotic-level Proteinuria event, Nephrotic Syndrome event, Proteinuria event, Renal event, and Steroid use
Abbreviations: CKD, chronic kidney disease; CVD, cardiovascular disease; PPPM, per-patient-per-month

Supplemental Table 3: Estimated CVD and Nephrotic Syndrome Event Rates in Adults

	Event Type	Patients	Events	Person Years	Rate (per 100 PY)	95% CI
**Baseline proteinuria ≤1.5 g/g**	CVD event	687	224	2701	8.3	7.3-9.5
	Nephrotic Syndrome event	687	622	2701	23.0	21.3-24.9
**Baseline proteinuria >1.5 g/g**	CVD event	1131	918	3873	23.7	22.2-25.3
	Nephrotic Syndrome event	1131	3,336	3873	86.1	83.3-89.1
**Baseline proteinuria <3.5 g/g**	CVD event	1172	524	4455	11.8	10.8-12.8
	Nephrotic Syndrome event	1172	1,460	4455	32.8	31.1-34.5
**Baseline proteinuria ≥3.5 g/g**	CVD event	646	618	2119	29.2	27.0-31.6
	Nephrotic Syndrome event	646	2,498	2119	117.9	113.4-122.6
**Post-KF**	CVD event	4792	3,004	15140	19.8	19.1-20.6

Abbreviations: CI, confidence interval; CVD, cardiovascular disease; KF, kidney failure; PY, person-years
